# Influence of delayed initiation of adjuvant chemotherapy on breast cancer survival is subtype-dependent

**DOI:** 10.18632/oncotarget.10551

**Published:** 2016-07-13

**Authors:** Ke-Da Yu, Lei Fan, Li-Xin Qiu, Hong Ling, Yi-Zhou Jiang, Zhi-Ming Shao

**Affiliations:** ^1^ Department of Breast Surgery, Shanghai Cancer Center and Cancer Institute, Shanghai Medical College, Fudan University, Shanghai, P.R. China; ^2^ Department of Medical Oncology, Shanghai Cancer Center and Cancer Institute, Shanghai Medical College, Fudan University, Shanghai, P.R. China

**Keywords:** timing of adjuvant chemotherapy, breast cancer, survival, subtype

## Abstract

**Purpose:**

The optimal time from surgery to initiation of adjuvant chemotherapy of breast cancer is still controversial. We investigated the influence of time to adjuvant chemotherapy on survival outcomes according to breast cancer subtype.

**Results:**

Longer delay of initiation of adjuvant chemotherapy (≤4 weeks versus >8 weeks)) significantly decreased the DFS (adjusted hazard ratio [HR] of 1.86; 95% confidence interval [CI], 1.19-2.90) and OS (adjusted HR of 2.02; 95% CI, 1.10-3.71). However, a moderate delay (≤4 weeks versus 4-8 weeks) did not significantly influence the survival. We further investigated the effect of time to adjuvant chemotherapy (≤8 versus >8 weeks) on survival according to subtypes. Patients with luminal-A tumors who received delayed chemotherapy had no increased risk of recurrence (HR of 1.15; 95% CI, 0.54-2.43). In contrast, patients with luminal-B, triple-negative, or trastuzumab-untreated HER2-positive tumors would have decreased DFS because of delayed chemotherapy, with HR of 1.93 (95% CI, 1.10-3.34), 2.55 (95% CI, 1.25-5.18), and 2.41 (95% CI, 1.36-4.26), respectively.

**Methods:**

Operable women with stage I-IIIa breast cancer between 2003 and 2006 in our institution were included. 1,408 patients were divided into 3 groups according to the time to adjuvant chemotherapy: ≤4 weeks, 4-8 weeks, and >8 weeks. Disease-free survival (DFS) and overall survival (OS) were calculated.

**Conclusion:**

Longer delay of adjuvant chemotherapy was associated with worse survival and early initiation of adjuvant chemotherapy should be performed for patients with aggressive tumor subtypes.

## INTRODUCTION

Meta-analysis of the adjuvant chemotherapy randomized controlled trials has demonstrated that adjuvant chemotherapy could decrease 30-40% risk of death caused by breast cancer versus those without chemotherapy [1]. At present, adjuvant chemotherapy is routinely recommended to 60-70% of breast cancer patients after surgery.

However, the optimal time from surgery to initiation of adjuvant chemotherapy is still controversial. In 1970s, investigators found that tumor removal as well as surgical trauma might lead to an accelerated growth of distant micrometastasis [2], providing a biological rationale to start chemotherapy as soon as possible after the removal of the primary tumor. However, it seems that it is unrealistic to conduct a randomized trial of time to adjuvant chemotherapy. Several retrospective studies evaluating the role of early or delayed initiation of adjuvant chemotherapy have been published reporting conflicting results [3–7]. It is could be explained by the differences in characteristics of patients and diseases, as well as the arbitrarily selected cutoff for delayed time.

We previously performed a systematic review and meta-analysis [8]. Seven eligible studies involving 34,097 patients (studies from 1978 to 2013) were included. Most used adjuvant chemotherapeutic regimens were cyclophosphamide, methotrexate, and fluorouracil (CMF) or anthracycline-based. Our meta-analysis demonstrated that a 4-week delay of chemotherapy was associated with a significant decrease in both overall survival (OS)(hazard ratio [HR], 1.15; 95% confidence interval [CI], 1.03-1.28) and disease-free survival (DFS)(HR, 1.16; 95% CI, 1.01-1.33). Since most included studies used CMF and anthracycline-based regimen, whether the results could extrapolate to the current taxane-based therapy is unknown. Moreover, a most recent report from Gagliato et al [9] indicates that the delayed adjuvant chemotherapy is particularly meaningful for patients with advanced disease, triple-negative breast cancer (TNBC), and trastuzumab-treated HER2-positive (HER2+) tumors. Whether the influence of delayed chemotherapy on breast cancer survival is subtype-dependent or not needs further investigation. Therefore, we conducted this retrospective analysis using our single-institution data to provide a complementary piece of evidence in this field.

## RESULTS

**Figure 1:**
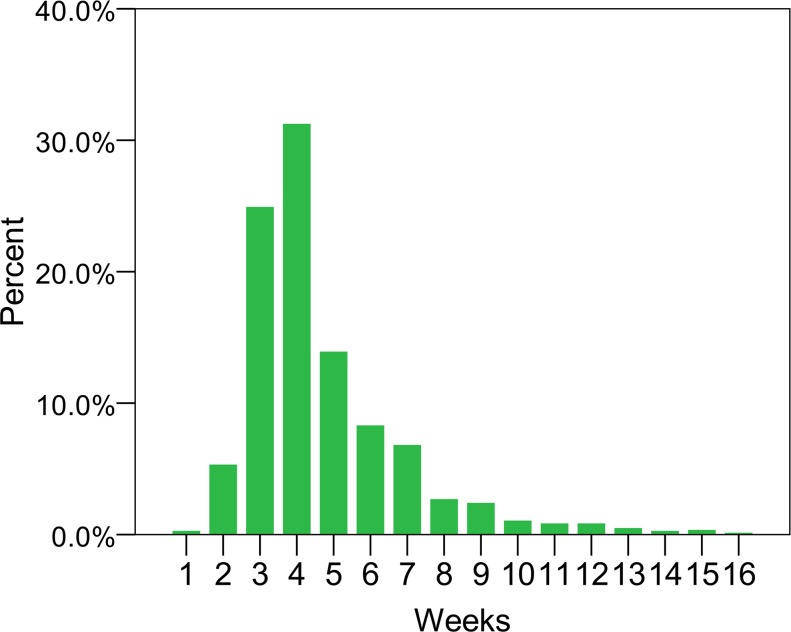
The distribution of patients by interval from surgery to the start of adjuvant chemotherapy

In the present study, the association between staring time of adjuvant chemotherapy and survival in 1,408 operable breast cancer patients was investigated. About 60% of patients received their adjuvant chemotherapy initiated within 4 weeks of surgery, and a minority (6.5%) started adjuvant chemotherapy after 8 weeks from surgery (Figure 1). Most of the clinical and pathological factors were equally distributed between the three groups by time of adjuvant chemotherapy (Table 1). However, women with delayed adjuvant chemotherapy were likely to have older age and indolent disease with negative nodes. The 5-year DFS were 80%, 78%, and 60% (log-rank P<0.001) and 5-year OS were 90%, 86%, and 81% (log-rank P=0.021) for group 1 (start chemotherapy ≤4 weeks), group 2 (4-8 weeks), and group 3 (>8 weeks), respectively.

**Table 1:** Patient characteristics by interval from surgery to adjuvant chemotherapy

Characteristics	Interval from surgery to chemotherapy initiation (weeks)								
	Total (n=1,408)		0-4 (n=871)		4-8 (n=446)		>8 (n=91)		P^#^
	n	%	n	%	n	%	n	%	
Median age, years		50		50		49		51	0.02
Tumor size, cm									
≤2	383	28.5	244	29.2	114	27.1	25	28.7	0.31
2-5	847	63.0	522	62.4	275	65.5	50	57.5	
>5	114	8.5	71	8.5	31	7.4	12	13.8	
Number of positive lymph nodes									
0	493	40.0	320	41.8	133	34.8	40	48.2	0.02
1-3	389	31.6	248	32.4	122	31.9	19	22.9	
≥4	349	28.4	198	25.8	127	33.2	24	28.9	
Surgical modality									
MS	1,341	95.4	828	95.3	424	95.3	89	97.8	0.54
BCS	64	4.6	41	4.7	21	4.7	2	2.2	
Endocrine therapy									
No	687	49.3	421	48.7	229	51.9	37	41.6	0.18
Yes	707	50.7	443	51.3	212	48.1	52	58.4	
Molecular subtype									
Luminal-A	667	47.4	431	49.5	191	42.8	45	49.5	0.08
Luminal-B	328	23.3	188	21.6	120	26.9	20	22.0	
TNBC	270	19.2	155	17.8	95	21.3	20	22.0	
HER2+	143	10.2	97	11.1	40	9.0	6	6.6	

Abbreviations: BCS, breast conservative surgery; MS, mastectomy; TNBC, triple-negative breast cancer

Some cases had missing data; the number of missing cases is not shown in the table.

^#^P value for different distribution in 3 groups is tested by heterogeneous x^2^.

**Figure 2:**
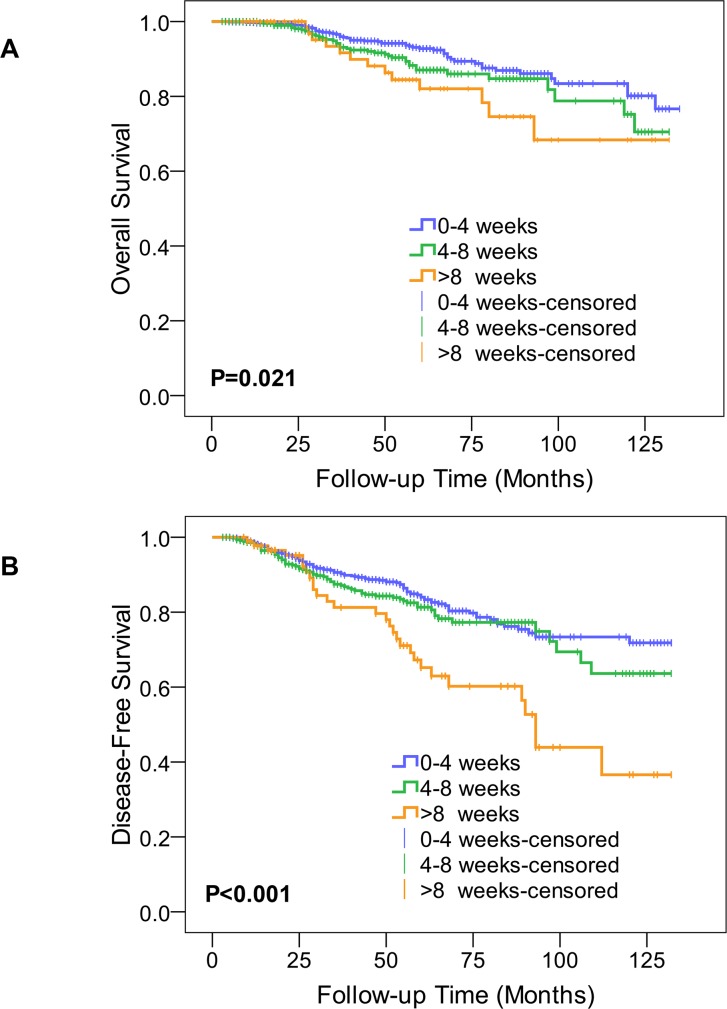
Kaplan-Meier plot for overall survival and disease-free survival according to interval between surgery and initiation of adjuvant chemotherapy 2A shows the Kaplan-Meier curves for overall survival for the three groups: 0 to 4 weeks, 4 to 8 weeks, and longer than 8 weeks from definitive surgery to start of adjuvant chemotherapy. 2B is for disease-free survival.

In the univariate analysis, DFS was significantly better for group 1 and group 2 compared with the group 3, and the numerical differences between group 1 and group 2 were not statistically significant (log-rank P=0.198 for group 1 vs. 2; P<0.001 for group 1 vs. 3; P=0.002 for group 2 vs. 3). Similar outcomes were found in OS analysis (log-rank P=0.084 for group 1 vs. 2; P=0.002 for group 1 vs. 3; P=0.049 for group 2 vs. 3). Kaplan-Meier curves for DFS and OS are shown in Figure 2A and 2B, respectively.

In the multivariate analysis, independent prognostic factors for DFS were tumor size, nodes status, adjuvant endocrine therapy, molecular subtype, and time to adjuvant chemotherapy (Table 2). A long delay of initiation of adjuvant chemotherapy at more than 8 weeks significantly decreased the DFS (HR of 1.86; 95% CI, 1.19-2.90) and OS (HR of 2.02; 95% CI, 1.10-3.71) after adjusting other prognostic factors. Interestingly, a moderate delay did not significantly compromise the survival (≤4 vs. 4-8 weeks: HR for DFS, 1.14, 95% CI, 0.83-1.56; HR for OS, 1.43, 95% CI, 0.94-2.19).

**Table 2:** Prognostic factors for disease-free survival and overall survival in multivariate analysis

Factor		Disease-free survival			Overall survival		
		HR*	95.0% CI	P^#^	HR*	95.0% CI	P^#^
Age	(continuous)	0.96	0.72-1.28	0.77	0.93	0.63-1.38	0.73
Size	(<2cm vs. 2-5cm)	1.41	0.92-2.14	0.11	2.22	1.10-4.47	0.03
	(<2cm vs. >5cm)	1.94	1.16-3.26	0.012	3.03	1.35-6.80	0.007
Nodal status	(0 vs. 1-3)	1.88	1.21-2.92	0.005	1.68	0.89-3.20	0.11
	(0 vs. ≥4)	5.38	3.66-7.91	<0.001	6.24	3.66-10.64	<0.001
Surgical modality	(Mast. vs. BCS)	0.56	0.21-1.49	0.24	0.78	0.29-2.11	0.63
Endocrine therapy	(No vs. Yes)	1.24	0.80-1.94	0.34	1.98	0.99-3.96	0.05
Molecular subtype	(luminal-A vs. -B)	1.35	0.91-2.01	0.14	1.13	0.63-2.06	0.67
	(luminal-A vs. TNBC)	2.30	1.38-3.84	0.001	3.59	1.68-7.66	0.001
	(luminal-A vs. HER2+)	2.32	1.30-4.12	0.004	3.74	1.63-8.61	0.002
Time to chemotherapy	(0-4 vs. 4-8 weeks)	1.14	0.83-1.56	0.42	1.43	0.94-2.19	0.09
	(0-4 vs. >8 weeks)	1.86	1.19-2.90	0.006	2.02	1.10-3.71	0.02

Abbreviations: BCS, breast conservative surgery; CI, confidence interval; HR, hazard ratio; MS, mastectomy; TNBC, triple-negative breast cancer

Method for Cox proportional hazards analysis: Enter all the variables.

^#^Adjusted for all the factors listed in the table.

Subsequently, we investigated the effect of time to chemotherapy (≤8 weeks vs >8 weeks) on survival according to breast cancer subtypes. In the univariate analysis, patients receiving adjuvant chemotherapy more than 8 weeks from surgery had a worse DFS than those receiving at an earlier time in luminal-B (P=0.016), TNBC (P=0.007), and trastuzumab-untreated HER2+ tumors (P<0.001), but not in luminal-A tumors (P=0.47) (Table 3). After adjustment for age, tumor size, nodal status, surgical modality, and adjuvant endocrine therapy, the results remained. Among patients with luminal-A disease, delayed chemotherapy had limited influence on recurrence (HR=1.15; 95% CI, 0.54-2.43). In contrast, patients with luminal-B, TNBC, and trastuzumab-untreated HER2+ tumors who received delayed chemotherapy had a HR of 1.93 (95% CI, 1.10-3.34), 2.55 (95% CI, 1.25-5.18), and 2.41 (95% CI, 1.36-4.26) for DFS, respectively, when compared with those started chemotherapy earlier (Table 3).

**Table 3:** Influence of time to adjuvant chemotherapy on disease-free survival in different subtypes

Subtype	Hazard for time to adjuvant chemotherapy (≤8 vs. >8 weeks)		
	Univariate P (log rank)	Multivariate HR (95% CI)^#^	Multivariate P
Luminal-A (n=667)	0.47	1.15 (0.54-2.43)	0.72
Luminal-B (n=328)	0.016	1.93 (1.10-3.34)	0.020
TNBC (n=270)	0.007	2.55 (1.25-5.18)	0.010
HER2+ (n=143)	<0.001	2.41 (1.36-4.26)	0.009

^#^Adjusted for age, tumor size, nodal status, surgical modality, and endocrine therapy.

CI, confidence interval; HR, hazard ratio; TNBC, triple-negative breast cancer

## DISCUSSION

The exact time window of adjuvant chemotherapy to gain maximal survival benefit is controversial. The previously publishedclinical trials do not specifically suggest the timing of chemotherapy after surgery. There is a wide variation across trials in the allowed time between surgery and adjuvant chemotherapy. In the present retrospective study, we observed that the time to adjuvant chemotherapy could influence survival outcomes for some specific molecular subtypes. Our study suggests that patients with biologically aggressive tumors would result in worse outcomes if the adjuvant chemotherapy was delayed.

In 1982, a report from M.D. Anderson hospital showed that there was no trend for longer delay in treatment to be associated with shorter DFS according to the length of delayed time (<10 weeks, 10-13, 14-17, and ≥18 weeks). However, poor prognosis patients with >14 weeks delays had significantly shorter disease-free interval [10]. Another analysis of the International (Ludwig) Breast Cancer Study Group Trials showed that, early initiation of systemic chemotherapy might improve outcome in premenopausal patients with ER-negative breast cancer but not in those with ER-positive tumors [7]. In the era of molecular subtype, a recent study indicated that delayed chemotherapy was associated with worse outcomes among patients with TNBC and trastuzumab-treated HER2+ patients [9], suggesting that a delay in the initiation of adjuvant chemotherapy could be dangerous for patients with aggressive subtypes.

Our results are consistent with the above-mentioned retrospective studies. Our findings suggest that triple-negative and HER2+ tumors should start adjuvant chemotherapy at an earlier time. In our study, no patient with HER2+ tumor receives trastuzumab. Those trastuzumab-untreated HER2+ patients would experience a significantly increased risk of recurrence when adjuvant chemotherapy was delayed. However, this phenomenon was not observed in the report from M.D. Anderson Cancer Center, in which the trastuzumab-treated HER2+ patients, but not the trastuzumab-untreated HER2+ patients, experienced worse outcomes when chemotherapy was delayed [9]. We believe that chemotherapy is a key component of the systemic treatment for HER2+ patients. Previous reports have demonstrated that HER2+ tumors is more sensitive to paclitaxel or anthracycline than HER2-negative tumors [11, 12] and our results imply that early initiation of chemotherapy is also important for patients with HER2+ tumors when trastuzumab is unavailable.

In addition, we found that luminal-B, rather than luminal-A, had a better survival if treated with chemotherapy without delay. A great number of trials have demonstrated that the magnitude of benefit of neoadjuvant chemotherapy in luminal-A is less pronounced than that in luminal-B tumors [13]. Moreover, luminal-A tumors could be more sensitive to endocrine therapy (which starts after adjuvant chemotherapy and lasts for 5 to 10 years) rather than chemotherapy. The updated 2015 St Gallen consensus indicates that chemotherapy might not be recommended for the majority of patients with luminal-A disease [14].

Our study has some limitations. First, this study is limited by the nonrandomized and retrospective nature. Our results need further validation in other population. Actually, the cycle number of chemotherapy, completion rate for adjuvant chemotherapy, as well dose reduction, were all the key determinants of survival, and should be adjusted. However, the retrospective data could not collect sufficient information. Second, some subtypes such as TNBC have small number size of samples and the results are statistically underpowered. Moreover, longer follow-up time is needed, particularly for patients with luminal-A tumors, who had a recurrence peak at 5 years after surgery rather than at 2 to 3 years post-surgery [15].

In conclusion, we demonstrated that a longer delay in the initiation of adjuvant chemotherapy was associated with worse survival outcomes. The influence varies according to subtypes and an early initiation of adjuvant chemotherapy is particularly important for patients with luminal-B, TNBC, and HER2+ tumors.

## MATERIALS AND METHODS

### Patients

We conducted a retrospective review of the Breast Surgery Department database at Fudan University Shanghai Cancer Center (FUSCC). The information of this database has been described previously [16, 17]. This study was approved by the Ethical Committee of the Shanghai Cancer Center of Fudan University, and each participant signed an informed consent document.

During 2003 to 2006, there were 2,203 consecutive patients who were diagnosed as stage I-IIIa breast cancer and treated surgically in FUSCC. Patients with stage IV disease are generally treated with palliative chemotherapy and were excluded from this study. The preoperative examination, surgical treatment, pathological evaluation, and adjuvant treatment strategy had been described in detail elsewhere [16]. Among them, 639 were excluded for following reasons: 451 receiving no adjuvant chemotherapy, 65 having neoadjuvant chemotherapy, and 123 receiving old regimen (e.g., CMF) or oral chemotherapeutic regimen only (e.g., capecitabine). The chemotherapeutic regimens used in this study were mainly anthracycline-based or anthracycline/taxane-based. None of the HER2+ patients received adjuvant anti-HER2 therapy such as trastuzumab. For the remaining 1,564 cases, 156 patients were further excluded because of short follow-up time (less than 6 months) or insufficient cycle number of chemotherapy (less than 4 cycles), and the final 1,408 patients made the analysis cohort. They were divided into 3 groups according to the time from surgery to adjuvant chemotherapy: less than or equal to 4 weeks (group 1, n=871), 4-8 weeks (group 2, n=446), and more than 8 weeks (group 3, n=91). Table 1 displays the basic patient characteristics. Because data for tumor grade were lacking in many cases, we did not include this variable in our analysis. Tumor stage could be judged by tumor size and lymph node status which were shown in Table 1.

Determination of estrogen receptor (ER), progesterone receptor (PR), and HER2 status was done by pathologists in the Department of Pathology in FUSCC. Positive ER or PR required that equal to or more than 10% of tumor cells are immunoreactive [18]. Patients with equivocal HER2 protein expression (immunohistochemistry 2+) had a FISH test for HER2 gene amplification [19]. This was done according to standard procedures [18, 19]. Molecular subtype was categorized as following [20]: luminal-A = ER+ or PR+, HER2-, and Ki67 < 14% (grade 1 or 2 if Ki67 index unavailable); luminal-B = ER+ or PR+, and HER2+ or Ki67≥14% (grade 3 if Ki67 unavailable [21]); HER2-enriched (HER2+) = ER-, PR-, and HER2+, any Ki67; and triple-negative breast cancer (TNBC) = ER-, PR-, HER2-, any Ki67.

### Survival analysis and statistics

The outcomes of interest were DFS and OS. DFS was calculated from surgery to the first event of relapse (local, regional, and/or distant), contralateral invasive breast cancer, or death in the absence of relapse. OS was calculated from surgery to death from any cause. All the patients should be followed at least for 6 months to maximize the reliability of vital status and recurrence data. The median follow-up time was 72 months (ranging from 6 to 132 months). DFS and OS curves were constructed using Kaplan-Meier method and compared by log-rank test. Multivariate analysis was performed with Cox proportional hazards analysis. Two-sided P<0.05 was considered statistically significant. Statistical analysis was performed using Stata v.14.0 (Stata Corporation, College Station, TX) and SPSS 17.0 (SPSS Inc, Chicago, IL).
